# Breastfeeding and Water Security

**DOI:** 10.1111/mcn.70114

**Published:** 2025-09-16

**Authors:** Rafael Pérez‐Escamilla, Victoria Hall Moran

**Affiliations:** ^1^ Department of Social and Behavioral Sciences Yale School of Public Health New Haven CT USA; ^2^ Maternal, Paternal and Infant Nutrition and Nurture Unit (MAINN) University of Lancashire PR1 2HE

Water security has been defined by the United Nations as “*The capacity of a population to safeguard sustainable access to adequate quantities of acceptable quality water for sustaining livelihoods, human well‐being, and socioeconomic development, for ensuring protection against water‐borne pollution and water‐related disasters, and for preserving ecosystems in a climate of peace and political stability”* (United Nations 2013). As such, water security is crucial for human and planetary health and the development of nations. As addressed at the 2025 World Water Week held in Stockholm August 24‐28, climate change has become a formidable challenge for water security globally. In this editorial we make the case that investing in breastfeeding protection, promotion and support should be a key component of national and global strategies to address water insecurity.

Global recommendations call for infants to be exclusively breastfed for 6 months, making breastmilk is the only source of water for infants of this age. As over 85% of breastmilk is water, babies can indeed meet their water needs through breastfeeding (Martin et al. 2016). Once complementary foods are introduced at around 6 months infants are recommended to continue to breastfeed for at least their first 2 years of life, hence breast milk can also be an important source of water security for infants and toddlers beyond the first 6 months of life.

It is estimated that 2,562.5 billion liters of water are used every year in the production and use of commercial milk formula (CMF) by infants under 6 months (Smith et al. 2024). Therefore, investing in breastfeeding protection, promotion and support can reduce substantially the use of water across the globe. Climate change mitigation requires bringing down the levels of greenhouse gases (GHG) which mainly come from industrial activity. The CMF industry generates between 5.9 and 7.5 billion kg CO2 eq. every year because of environmental degradation and fossil fuel use related to dairy farming, milk processing, and CMF production, transportation, storage and preparation. Exclusive breastfeeding, in contrast, produces a far lower carbon footprint compared to feeding with CMF (Andresen et al. 2022; Karlsson et al. 2019). The massive impact of the CMF industry on climate change and water use is evident at the country level. Using India as an example, CMF consumption among infants under 6 months utilizes 250.6 billion liters of water and GHG emissions range from 579 to 737 million kg CO2 eq. annually, despite the country's high breastfeeding prevalence among infants under 6 months (Smith et al. 2024). For this reason, it has been recommended for breastfeeding to be included in climate change mitigation agreements and investments within countries and across the globe (Smith et al. 2024) (Figure 1).

Moreover, climate change is driving an increase in extreme weather events such as droughts, floods, and storms, which frequently damage water and sanitation infrastructure. These disruptions intensify risks of food insecurity and waterborne diseases, leaving infants and young children especially vulnerable. In such circumstances, breastfeeding offers critical protection by providing a safe, nutritionally adequate, and pathogen‐free food source, while also conferring immunological benefits that reduce infection risk. Yet, disaster conditions often compromise breastfeeding through disrupted health systems, unregulated distribution of breast‐milk substitutes, and limited privacy in shelters ‐ factors that underscore the urgent need to prioritise and sustain breastfeeding as a core public health intervention during both emergency response and recovery phases (Bartick et al. 2024).

There is a profound connection between water security and food security (Young et al. 2021) and breastfeeding is a prime example of this relationship in early infancy. Although breastfeeding has been increasingly recognized as a key component of food systems under regular circumstances and during humanitarian emergencies (Pérez‐Escamilla and Moran 2023) it is rarely mentioned as being key for the water security of infants and young children and the planet. The contributions that women make to environmental protection through breastfeeding should be recognized through investments in strengthening the breastfeeding protection and support systems they need (Smith et al. 2024). There is ample evidence to support funding breastfeeding interventions and policies as legitimate carbon offsets investments (Smith et al. 2024). Moving forward MCN would like to encourage the submission of articles for consideration focusing on the role that breastfeeding has in the food and water security of infants, their families and societies, and the planet (Figure 1).

**Figure 1:**
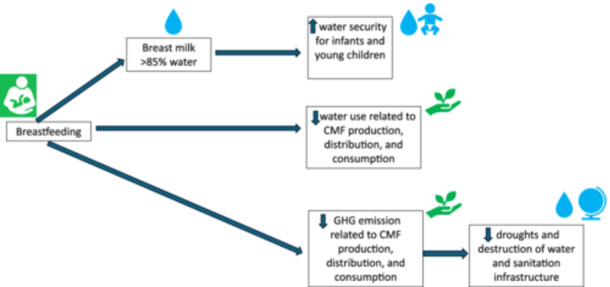
Pathways through which breastfeeding can improve water security among infants and young children and in the general population across the globe.

## Conflicts of Interest

The authors declare no conflicts of interest.
